# The Application of Embelin for Cancer Prevention and Therapy †

**DOI:** 10.3390/molecules23030621

**Published:** 2018-03-09

**Authors:** Jeong-Hyeon Ko, Seok-Geun Lee, Woong Mo Yang, Jae-Young Um, Gautam Sethi, Srishti Mishra, Muthu K. Shanmugam, Kwang Seok Ahn

**Affiliations:** 1 College of Korean Medicine, Kyung Hee University, 24 Kyungheedae-ro, Dongdaemun-gu, Seoul 02447, Korea; gokjh1647@gmail.com (J.-H.K.); seokgeun@khu.ac.kr (S.-G.L.); wmyang@khu.ac.kr (W.M.Y.); jyum@khu.ac.kr (J.-Y.U.); 2 Department for Management of Science and Technology Development, Ton Duc Thang University, Ho Chi Minh City 700000, Vietnam; 3 Faculty of Pharmacy, Ton Duc Thang University, Ho Chi Minh City 700000, Vietnam; 4 Department of Pharmacology, Yong Loo Lin School of Medicine, National University of Singapore, Singapore 117600, Singapore; srishti.mishra@u.nus.edu (S.M.); phcsmk@nus.edu.sg (M.K.S.)

**Keywords:** Embelin, apoptosis, autophagy, cancer, cell signaling

## Abstract

Embelin is a naturally-occurring benzoquinone compound that has been shown to possess many biological properties relevant to human cancer prevention and treatment, and increasing evidence indicates that embelin may modulate various characteristic hallmarks of tumor cells. This review summarizes the information related to the various oncogenic pathways that mediate embelin-induced cell death in multiple cancer cells. The mechanisms of the action of embelin are numerous, and most of them induce apoptotic cell death that may be intrinsic or extrinsic, and modulate the NF-κB, p53, PI3K/AKT, and STAT3 signaling pathways. Embelin also induces autophagy in cancer cells; however, these autophagic cell-death mechanisms of embelin have been less reported than the apoptotic ones. Recently, several autophagy-inducing agents have been used in the treatment of different human cancers, although they require further exploration before being transferred from the bench to the clinic. Therefore, embelin could be used as a potential agent for cancer therapy.

## 1. Introduction

**Figure 1:**
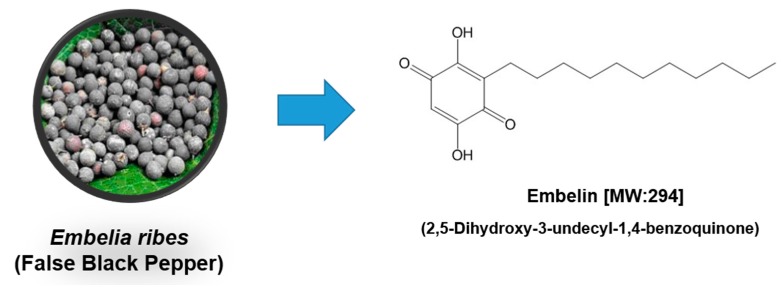
The chemical structure of embelin.

Embelin (2,5-dihydroxy-3-undecyl-1,4-benzoquinone) is a major active constituent obtained from the *Embelia ribes* Burm plant (specifically, its fruit), and has been evaluated for treating a range of cancer types [1] (Figure 1). Embelin is a non-toxic compound and is well tolerated up to a dose of 3 g/kg b.w. No subacute oral toxicity was detected after 10 mg/kg of embelin was administered to rats for 10 weeks [2]. A short-term toxicity study of embelin showed that oral administration of it to cyclic female rats for six weeks at 120 mg/kg was not toxic overall; however, some increase in the levels of alkaline and acid phosphatase was observed [3]. When embelin was administered for 14 weeks at 50 mg/kg b.w., it was found to be toxic to hematopoietic cells [4]. However, in contrast, when emeblin was administered to monkeys, rats, and mice for six months, it was found to be non-toxic, and no hematological toxicity was observed [5]. In addition, acute toxicity studies of mice that had been administered oral doses of 50 and 100 mg/kg of embelin revealed no notable change in mortality and body weight [6]. The toxicity of embelin was also assessed in a study involving pancreatic-cancer xenograft mice, after the mice spent six weeks on a 450 mg/kg diet. Blood analyses showed normal liver and kidney function in embelin dietary groups, suggesting that embelin was well tolerated and did not induce apparent toxicity [7]. The pharmacokinetics of the intravenous and oral administration of potassium embelate (20 mg/kg) to rats revealed that the compound followed a biexponential kinetic sequence. The absorption was fast and complete (the bioavailability was 97%), with the drug peak plasma level at 9 μg/mL at 0.28 h. The disposition half-life was 11 h for oral administration and 9.5 h for intravenous injection [8]. In another study, the oral administration of 50 mg/kg of embelin to rats resulted in 130.39 ± 6.51 μg/mL peak plasma concentration after 4.285 h [9]. The oral administration (70 mg/kg) of embelin to mice yielded its maximum concentration in plasma (3.55 ± 0.13 μg/mL) 1 h after administration, but it then declined rapidly to 0.26 ± 0.06 μg/mL at 3 h [7]. According to Gupta et al. [10] oral administration (75 mg/kg/day) of embelin for 30 days in rats resulted in the highest levels being in the kidneys, followed by the testes and intestines. Significant levels were observed in the brain, heart, and spleen. The level of embelin in all the organs was higher than after 15 days of treatment. The levels slowly declined in all of the organs after 30 days of embelin treatment, indicating slow elimination from the body. These results indicate that the possible accumulation of embelin in the tissues is one reason for its effective biological activities.

Numerous studies have determined the multiple biological properties of embelin, including anti-tumor, anti-inflammatory, analgesic, antidiabetic, and anti-oxidant effects [11,12,13,14]. A thorough computational-database screening revealed that, structurally, embelin could be characterized as a pharmacological inhibitor of XIAP (X chromosome–linked inhibitor-of-apoptosis protein) [15]. Embelin’s anti-cancer properties have been observed in in vivo experiments in rodents (Table 1). Furthermore, several studies have evaluated embelin’s role in anti-cancer combination therapy, highlighting the application of embelin in improving the efficacy of the treatment (Table 2). The anti-cancer activity of embelin is predominantly mediated by the modulation of various oncogenic transcription factors, inflammatory cytokines, growth factors, and protein kinases [16,17,18,19]. Strategically targeted cancer therapies are focused on investigating mechanisms associated with cell-growth and cell-death pathways. Imbalances in the coordination of cell proliferation and cell death can result in diseases such as cancer due to unrestrained cell growth and reduced cell death [20,21,22,23]. Apoptosis is the best-characterized and most evolutionarily conserved form of programmed cell death. It is a physiological phenomenon and is characterized by nuclear shrinkage, condensation and subsequent fragmentation, membrane blebbing, and formation of apoptotic bodies [21,24,25,26]. Resistance to apoptosis is a common phenomenon in numerous varieties of cancer, and is also the crucial element in tumorigenesis and therapy resistance [25,27,28,29]. Apoptotic pathways are, thus, legitimate focus areas for cancer therapy; predominantly natural agents like embelin can affect various cell-death pathways [28].

Autophagy, unlike apoptosis, is not synonymous with cell death, however. Autophagy is a catabolic process, highly conserved, of elimination of proteins and cellular organelles, and it includes macroautophagy, microautophagy, and chaperone-mediated autophagy [30,31]. Autophagy is also a process of cleansing the cells of unwanted cellular proteins by packaging them to lysosomes for degradation. Autophagy plays an important role in several physiological and pathological processes, such as cell survival, cellular metabolism, immunity development, and aging [31,32]. Autophagy accompanied by non-apoptotic cell death has been described in cancer cells [31,33,34]. Therefore, the induction of autophagy is as an attractive goal for cancer treatment and prevention. The induction of autophagic cell death and apoptosis as mechanisms of cancer prevention by embelin will now be discussed.

## 2. Apoptosis-Pathway Induction by Embelin in Cancer

### 2.1. Extrinsic Pathway

Embelin has been reported to modulate the extrinsic apoptotic pathway and inhibit TNF-α, TNF receptor-1, and TRADD gene expression [16]. Human breast-cancer cells treated with embelin have attenuated levels of TNF-α and the TNF-α-converting enzyme [19]. TNF-related apoptosis-inducing ligand (TRAIL) is a part of the TNF superfamily, which is known to selectively induce apoptosis within cancer cells without being significantly toxic toward normal cells [45]. TRAIL is an apoptosis-inducing ligand, especially in tumor cells [46]. Several cancers have acquired resistance to interleukin-mediated apoptosis primarily because of reduced TRAIL-R1/-R2 expression, the over-expression of anti-apoptotic proteins like XIAP and c-FLIP, or elevated TRAIL decoy receptors TRAIL-R3/R4 [47]. Recent work has shown that embelin has the ability to restore the TRAIL sensitivity of cancer cells that are TRAIL-resistant. Embelin enhanced the TRAIL sensitivity by inhibition of XIAP in pancreatic cancer, nasopharyngeal carcinoma, and inflammatory breast-cancer cells. Embelin also sensitized malignant glioma cells to TRAIL-mediated apoptosis via down-regulating FLIP [48]. In A549 non-small-cell lung-cancer cells, embelin was shown to enhance TRAIL-mediated apoptosis via decreasing the levels of survivin, Bcl-2, and FLIP [49]. In addition, embelin sensitized human leukemia cells to TRAIL-induced apoptosis through the up-regulation of DR4 and DR5 [50]. These findings suggest that embelin can be applied in conjunction with other therapeutic modalities for cancer therapy.

### 2.2. Intrinsic Pathway

The intrinsic pathway is also known as the mitochondrial pathway because it depends on factors released from the mitochondria. This pathway is regulated by the Bcl2 family of proteins, including anti-apoptotic (e.g., XIAP, Mcl-1, Bcl-xL, and Bcl-2) and pro-apoptotic (e.g., Smac, Bak, Bid, and Bax) proteins [51]. The mitochondrial pathway is activated by intrinsic death stimuli, which causes cytochrome c to be released from the mitochondria and the apoptosome complex (consisting of caspase-9, cytochrome c, and the apoptotic protease activating factor (Apaf1)) to be formed. The apoptosome activates caspase-9 which, in turn, activates caspase-3 [52]. It has been reported that embelin significantly induces apoptosis in a range of cancer cells via the mitochondria-dependent apoptosis pathway. Embelin induced apoptosis in human leukemia cells, which was mediated by the activation of caspase-dependent mechanisms that involved the down-regulation of XIAP [53]. XIAP is an important member of the inhibitors of apoptosis (IAP) family that is involved in cancer-cell survival [41,54]. XIAP inhibits both caspase-9 and -3, leading to suppression of apoptosis within cancer cells [55,56]. It has been shown that embelin promotes the release of cytochrome C thereby promoting the activation of PARP cleavage and executioner caspase-3 in cancer cells [57]. With MCF-7 breast-cancer cells, it was demonstrated that embelin promotes the mitochondrial release of cytochrome C, leading to the activation of caspase-9 and -3; there were no significant changes observed in the level of caspase-8 [58].

In brain glioma cells, embelin induced apoptosis and G0/G1-phase cell-cycle arrest. In addition, it modulated the shifting of Bcl-2 and Bax to prompt the mitochondrial release of cytochrome c, thereby activating the caspase proteins to induce apoptosis [59]. Furthermore, embelin induced the expression and oligomerization of voltage-dependent anion channel 1, situated in the outer mitochondrial membrane, which can function as a significant part of the permeability-transition pore that could promote cytochrome c as well as apoptosis-inducing factor release [60]. The apoptotic effects of embelin were also investigated in human acute T cell lymphoma Jurkat cells. Cell treatment with embelin resulted in the cleavage of PARP, the activation of caspase-3 and -9, a reduction in XIAP, Bcl-xL, and Bcl-2, and a concomitant rise in Bax [61].

### 2.3. Other Apoptotic Pathways

The discovery of NF-κB (nuclear factor kappa-light-chain-enhancer of activated B cells) was prompted by a preliminary observation by David Baltimore in 1986 that a ubiquitously-expressed protein NF-κB was able to bind a short immunoglobulin kappa-light-chain enhancer DNA sequence. Since its initial discovery, NF-κB has been heavily studied and identified as having roles in the regulation of apoptosis and survival signaling, pro- and anti-inflammatory reactions, memory formation, cell-adhesion maintenance, and many aspects of the immune response, including viral-infection responses. Indeed, its central role in immune-response control and the orchestration of cytokine and inflammatory signaling has led many to describe NF-κB as the prototypical proinflammatory signaling pathway [62,63,64,65,66,67,68]. Embelin was found to inhibit the activation of NF-κB induced by TNF-α and several other carcinogenic and inflammatory agents, including lipopolysaccharide, interleukin-1β, phorbol myristate acetate, hydrogen peroxide, cigarette-smoke condensate, and okadaic acid [16]. Embelin down-regulated the TNF-α-induced expression of various NF-κB-dependent anti-apoptotic proteins in KBM-5 chronic myelogenous leukemia cells [16]. It was found that apoptosis induced by embelin in human glioma was mediated via the inhibition of NF-κB activity, which in turn occurred via reduction of the nuclear translocation of p65 through reducing the phosphorylation and proteasomal degradation of IκB-α [69]. Embelin could also sensitize HL-60 acute myeloid leukemia cells to TRAIL through the inactivation of NF-κB in in vitro and in vivo HL-60 xenograft models [70].

Recently, it was shown that embelin could modulate several pathways linked to apoptosis and cell growth, such as JAK/STAT, PI3K/AKT, p53, and p38 MAPK in gastric-cancer cells [71,72]. Embelin also attenuated cell invasion and proliferation, and induced apoptosis via inhibiting STAT3 and activating p53 signaling pathways within mouse pancreatic-cancer cells [39]. The tumor-suppressor protein p53 regulates several cellular processes, like apoptosis, proliferation, cell cycle, genome integrity, and DNA damage response [73,74]. Once active, cytoplasmic p53 translocates to the nucleus and can bind itself to regulatory DNA sequences, and activate the expression of target genes, such as cell-cycle inhibition, angiogenesis, and induction of apoptosis [75,76,77]. p53 is an important pro-apoptotic factor and tumor inhibitor, and many natural chemopreventive agents have been found to trigger apoptosis and cell-cycle arrest by activating p53, as well as its target genes [21,26]. Embelin attenuated cell proliferation, blocked metastatic migration, regulated the expression of caspases and Bcl-2, and triggered apoptosis (which was found to be mediated by p53) within MCF-7 breast-cancer cells [78]. Furthermore, embelin was found to inhibit mortalin-p53 interactions. The binding of embelin with mortalin/p53 releases free p53, which then translocates to the nucleus and initiates gene transcription that augments the growth suppression of breast-cancer cells [79].

XIAP prevents apoptosis through phosphatidylinositol 3-kinase (PI3K)-dependent inhibition of the caspase cascade [80]. There is possible involvement of the PI3K/AKT survival pathway in XIAP-mediated chemoresistance of ovarian-cancer cells [80]. PI3K was first discovered as a lipid kinase that phosphorylates phosphoinositides (PtdIns) at position 3 of the inositol ring. Several studies have reported on the role of PI3K as a pro-survival signaling molecule that can be activated by growth factors [81]. Upon activation, PI3K phosphorylates AKT in a serine/threonine residue, thereby activating the PI3K/AKT pathway [82,83,84,85]. The phosphorylated AKT subsequently inactivates Bad, a pro-apoptotic element of the Bcl-2 group [86]. In PC-3 prostate-cancer cells, embelin prevented the AKT/mTOR/S6K1 signaling cascade from being constitutively activated, which corresponded with substantial apoptosis being triggered, as evidenced by various biochemical assays [18,72]. In addition, the suppression of GSK-3β activation and AKT signaling is partly responsible for embelin’s pro-apoptotic effect [60]. Embelin decreased the constitutive phosphorylation/activation levels of PI3K/AKT in bladder cancer, pancreatic cancer, and leukemia [57,87,88], and inhibited cell growth by inducing apoptosis via the PI3K/AKT pathway. When embelin was used in combination treatment with either an AKT inhibitor or a PI3K inhibitor (LY294002), downstream caspase-3 and -9 experienced activation and cleavage, and PARP experienced cleavage. These results clearly demonstrate that suppressing the PI3K/AKT pathway and inhibiting XIAP results in more efficient apoptosis in diffuse, large B-cell lymphoma [54]. The combination of LY294002 and embelin also resulted in the synergistic induction of apoptosis in papillary thyroid carcinoma and breast-cancer cells, and the regression of tumor growth in vivo [40,41]. Therefore, embelin alone or in combination with inhibitors of the PI3K/AKT pathway may have therapeutic utility in cancers with upregulated XIAP expression.

STAT3 (signal transducer and activator of transcription 3), part of a group of six varied transcription factors, has been reported to be closely linked with the survival characteristics and aberrant growth of tumor cells [89,90,91,92,93,94]. Embelin suppressed STAT3′s constitutive activation in several cancers, including human multiple myeloma (U266), head and neck squamous carcinoma (SCC4), and human prostate carcinoma (DU-145) [17]. Embelin also suppressed proliferation and augmented apoptosis dose-dependently within human pancreatic HPAF-II and MIA PaCa-2 cancer cells, and decreased the phosphorylation of STAT3 and the expression of surviving, its downstream target, within MIA PaCa-2 cells [7]. Furthermore, embelin dose-dependently suppressed the phosphorylation of STAT3 within mouse pancreatic H7 and Panc 02 tumor cells, both in vitro and in vivo [39,72].

## 3. Autophagy Pathway Induced by Embelin in Cancer

Autophagy is a conserved cellular process that regulates multiple physiological processes, is associated with several human ailments, and plays a pivotal role in self-defense against pathogen infections. Mechanistically, deregulated autophagy in cancer was reported in 1999, and more recently extensive attention has been paid to elucidating the paradoxical role of autophagy in tumor suppression and tumor promotion [95]. Autophagy is a genetically-programmed and evolutionarily-conserved catabolic pathway that induces a specific form of programmed cell death that is different from caspases-mediated apoptosis [96]. The persistent activation of autophagy also has a part in several biological processes, like cell growth and protein synthesis [96,97,98,99,100,101,102]. Embelin was shown to induce autophagy in human oral squamous carcinoma Ca9-22 cells. Beclin 1/Atg six is an important component in the formation of autophagic vesicles. The embelin-induced autophagic cell death of oral-cancer cells was mediated by LC3-II and the suppression of p62/SQSTM1 and Beclin-1, and was associated with cleavage formation of the Atg5-Atg12 complex and Beline-1 [1]. In addition, embelin’s anti-cancer activity was mediated by the concomitant induction of both autophagy and apoptosis [1].

**Figure 2:**
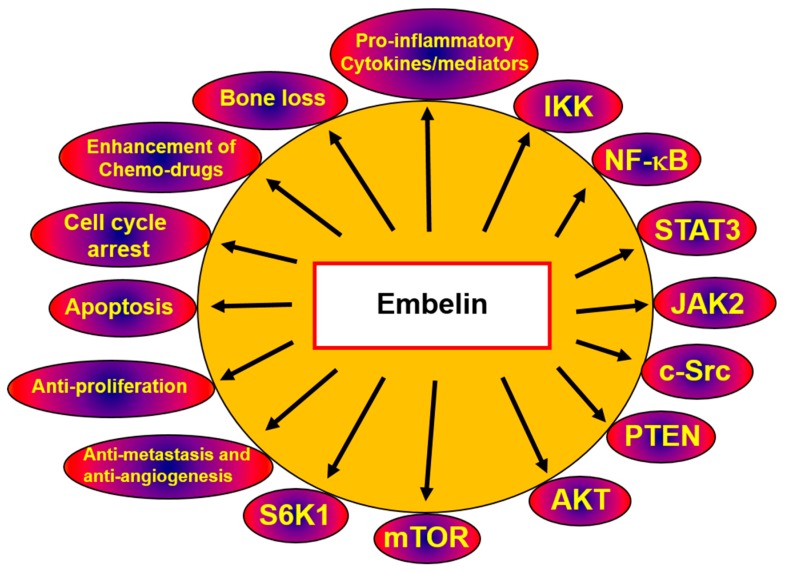
Proposed mechanisms of the potential anticancer activities of embelin.

Autophagy and apoptosis are two of the most important physiological processes. Despite the marked differences in their mechanisms of action, they are highly interconnected and perform complex cross-talk in inducing apoptosis. It has been observed that the pro-apoptotic signals activate caspase-3. Activated caspases also degrade autophagy proteins such as Beclin 1, Atg5, and Atg7 by downregulating the autophagy response [103]. When autophagy is inhibited, the apoptotic pathway takes over, displaying characteristic chromatin condensation, nuclear fragmentation, and membrane blebbing [104]. Thus, the deregulated apoptotic pathway is often found in cancer cells, and in low-stress conditions autophagy is initiated as a pro-survival mechanism. However, in cancer cells, autophagy-induced cell death has been observed as an alternative mode of action [104,105]. Cross-talk between autophagy and apoptosis has also been reported, from upstream regulators to the core machinery [104]. When designing anti-cancer drugs such as embelin, which target autophagic mechanisms, two different strategies can be adopted: either trigger autophagy and augment its tumor-suppression properties or suppress autophagy and then induce apoptosis [106]. Overall, the proposed mechanisms underlying the anti-cancer activities of embelin are depicted in Figure 2. In K562 and U937 leukemia cells, caspase inhibitor z-VAD-fmk reversed embelin-induced apoptosis, which suggests that apoptosis mediated by embelin is also partially mediated by caspases. In addition, embelin increased the expression of LC3-II in both K562 and U937 cells, indicating that embelin-induced cell death is associated with both autophagy and caspase-dependent apoptosis [57].

## 4. Redox- and Non-Redox-Dependent Actions of Embelin

Recent evidence suggests that the redox-modulating action of embelin is a mechanism responsible for the in vitro inhibition of COX. However, the redox mechanism on its own cannot account for embelin’s activity. Molecular-modeling studies have revealed similar binding modes that are also similar to nonsteroidal anti-inflammatory drugs (NSAIDs), which are extensively employed for the treatment of chronic and acute inflammatory disorders [107]. In silico modeling suggested that embelin would initiate a hydrogen bond together with Tyr355, while the alkyl chain would fit into a hydrophobic binding pocket that would form, resulting in Tyr385 [107]. In HT-29 colon-cancer cells, embelin increased the amount of lipid peroxides with concomitant depletion of the total amount of glutathione S-transferase and GSH (reduced glutathione) activity, thereby increasing oxidative stress and the induction of apoptosis [108]. Embelin induced cytotoxicity in HL-60 dose-dependently by increasing the generation of intracellular oxidation and the oxidative stress [109]. A dose of 50 mg/kg of embelin lowered lipid peroxidation, stopped the fall in the hepatic glutathione antioxidant defense, and reduced the histological alterations caused by chemically-induced hepatocarcinogenesis in Wistar rats [4]. In another study, increasing concentrations of embelin decreased the levels of lipid peroxide and glutathione in fibrosarcoma cells [110]. Embelin can sensitize inflammatory breast-cancer cells to TRAIL-mediated apoptosis via shifting the cellular redox balance, an indication that ROS modulators represent a novel approach to the treatment of inflammatory breast cancer [111]. Embelin may also be used with photodynamic therapy (PDT), which employs photochemical reactions mediated via interaction with a photosensitizing agent for treating malignant tumors. Embelin in combination with PDT increased ROS accumulation in Ehrlich ascites carcinoma cells and induced apoptosis [112]. Similarly, in A549 lung-cancer cells, embelin can induce ROS activation and subsequently lead to the induction of apoptosis [113]. Therefore, redox imbalance and pro-oxidant pathways may also contribute to embelin-induced cancer-cell death.

## 5. Embelin Derivatives Complex Functions as a Catalyst and as an Anti-Cancer Agent

Quinonic compounds are found ubiquitously in nature and are mainly involved the transfer of hydrogen and electrons and they also form a big group of anticancer agents that are either in the preclinical or clinical development [114,115]. Embelin is a 1,4-benzoquinolic compound that has been shown to exhibit significant anti-cancer activities. Several studies have indicated the unique and remarkable role of novel embelin complexes that display fairly good catalytic activity for the reduction of molecular oxygen. One of the earliest study reported on the synthesis and structure of transition metal complexes of embelin, such as complexes of Mn(II), Ni(II), Cu(II), and Zn(II) with embelin [116]. Encapsulation of copper (II) complexes with two ligands of embelin was catalytically active and greatly increased the oxygen binding ability of the metal ion. This finding has far reaching consequences in the applications of gas purification [117]. In another recent report, new derivatives of embelin that have aromatic groups linked to the benzoquinone core via the Suzuki–Miyaura reaction were synthesized and tested for its XIAP inhibitory activity in cancer cells [118]. The benzoquinone core of embelin has been utilized to synthesize O-CH3 and O-C2H5 groups. It was found that the embelin analogues possessed good pro-apoptotic activities against selected tumor cells [119]. A recent study by Evans et al. showed that embelin derivatives can be synthesized by Knoevenegal/Hetero-Diels–Alder domino reactions (DKHDA) [120,121] and reported that the promising structural backbone for the synthesis of bioactive drug candidates [72]. In addition, several novel benzyl-piperidine derivatives of embelin showed potent anti-proliferative activities against a variety of breast, prostate, pancreatic and colon cancer cells and induced mitochondria mediated apoptosis [122]. Novel hydrophilic analogues of embelin consisting of 3 amine groups with differences in the length of the carbon chain was found to be cytotoxic to KERATIN-forming tumor *cell* line HeLa KB cancer cells [123]. Several other new embelin derivatives were also reported to have potent XIAP inhibitory activity [124]. In another recent report, embelin-derived rhodium Rh(III) and iridium Ir(III) metalla-rectangles derivatives inhibited the growth of DU-145 prostate cancer, A-549 lung cancer, and cervical cancer HeLa cells. In contrast there was a minimal effect on the growth of non-cancerous HEK-293 cells [125].

## 6. Conclusions

Embelin, a naturally-occurring benzoquinone, exhibits many anti-cancer properties in various cancer cells. Embelin induces apoptosis by targeting several signaling pathways, which differ considerably based on the origin of the cancer. Tumor cells employ multiple survival pathways to promote their initiation, progression, and metastasis. Accordingly, agents such as embelin, which can suppress multiple cellular and redox pathways, may have strong potential for cancer prevention and treatment. Targeting autophagy in cancer may be a feasible approach in cancer therapy, and, since autophagy has a part in the suppression of tumors, the use of embelin-induced autophagy could be a significant strategy for cancer prevention.

## Figures and Tables

**Table 1:** In vivo anti-cancer effects of embelin.

Cancer Model	Animal Model	Dose	Outcome	References
	DMH models in male and female C57 mice	100 mg/d/kg body weight (b.w.) mixed in diet for 30 weeks	Tumor incidence↓, Tumor multiplicity↓, Cox-2↓; PCNA↓; c-Myc↓; Survivin↓	[35]
Colon	AOM/DSS induced coloncancer in male C57BL/6 mice	50 mg/d/kg b.w. mixed in diet for 10 days before the CACchallenge, then for 19 or 85 days	Tumor incidence↓, Tumor volume↓, IL-6↓;STAT3↓	[36]
Ehrlich’s ascites Carcinoma (EAC)	Male Swiss albino mice solid tumor model with EAC cells	Photodynamic therapywith Embelin 12.5 mg/kg b.w. i.p.	Tumor incidence↓, Tumor volume↓, Myeloperoxidase↓, β-d-glucuronidase↓, Rhodanese↑, Bcl-2↓; Bax↑	[37]
Liver	DENA/PB induced hepatocarcinogenesis in male Wistar rats	50 mg/kg b.w. per os(p.o.) for 14 weeks	Neoplastic nodules↓,	[38]
Pancreas	Female C57BL/6 Ectopic mouse model with H7 or Panc 02 cells Female C57BL/6 Orthotopic mouse model with H7 or Panc 02 cells	50 mg/kg b.w. *intraperitoneal injection* (i.p.) daily for two weeks 50 mg/kg b.w. i.p. every other day for one week	Tumor volume↓ Tumor volume↓ Metastasis↓	[39]

**Table 2:** Synergistic anti-cancer effects of embelin in vivo.

Cancer Model	Animal Model	Dose	Outcome	References
Breast	Female nude mice xenograft models of MDA-MB-231 cells	Embelin 10 mg/kg b.w. and LY294002 10 mg/kg b.w. i.p. twice weekly for four weeks	Tumor volume↓, XIAP↓; Bcl-2↓; Bxl-xL↓ AKT↓; caspase-3↓	[40]
Pancreas	Male athymic nude mice xenograft models of HPAF-II cells	Ellagic acid 150 mg/kg diet, daily 25 mg/kg b.w. and Embelin 450 mg/kg diet, daily 75 mg/kg b.w. for one week beforetumor implantation, andthen for five weeks	Tumor volume↓, Tumor cellularity↓	[7]
Papillary Thyroid Carcinoma	Nude mice xenograft with TPC1 cells	Embelin 10 mg/kg b.w.and LY294002 10 mg/kg b.w. i.p. twice weekly for four weeks	Tumor volume↓, XIAP↓; p-AKT↓; caspase-3 and -8↓; Bcl-2↓; Bxl-Xl↓	[41]
Prostate	Male Balb/c nude mice xenograft models of LNCaP cells	Bicalutamide 20 mg/kg three times a week and then embelin-loaded micellesintratumoral injection from day 28	Tumor volume↓	[42]
	Male athymic nu/nu mice xenograft models of C4-2 cells	CBDIV17 antiandrogen10 mg/kg and embelin 10 mg/kg-loaded micelles, intratumoral injection on days 0, 3, and 7	Tumor volume↓	[43]
	Female athymic NCr-nu/nu mice xenograft models of PC-3 cells	X-ray radiation at 2 Gy fraction on days 1 to 5 weekly for 2 weeks and Embelin 60 mg/kg p.o. on days1 to 5 weekly for three weeks	Tumor volume↓, Ki67 and PCNA↓, TUNEL↑; PARP cleavage↑, CD31↓	[44]
